# Supplementary material for: Predicting COVID-19 in very large countries: The case of Brazil
Source: PLoS One. 2021 Jul 1;16(7):e0253146. doi: 10.1371/journal.pone.0253146 (PMC8248665; doi:10.1371/journal.pone.0253146)
Supplement: Attachment — Submitted filename: Response to Reviewers.pdf [file pone.0253146.s001.pdf]

Response to the editor's and reviewer's comments

Date: January 2021

Dear Editor and reviewers,

Thank you for giving us an opportunity to revise our manuscript entitled " Predicting COVID-19 in very large countries: the case of Brazil ". We found the reviewers' comments/feedback very helpful in improving the manuscript and we have revised the manuscript accordingly. Please find attached the revised manuscript. This work was supported by Mauá Institute of Technology. We declare that all authors have no conflicts of interest. The manuscript has not been published in any other journal. There is a preprint version at: https://www.researchsquare.com/article/rs-79708/v1. Our point-by-point comments on the suggested revisions are below.

Best regards,

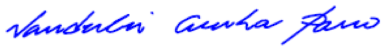

Vanderlei Cunha Parro (corresponding author)
Full professor
+ 55 11 4239 31021
+ 55 11 9 7513 4631

Journal Requirements

1. Please ensure that your manuscript meets PLOS ONE's style requirements, including those for file naming. The PLOS ONE style templates can be found at

https://journals.plos.org/plosone/s/file?id=wjVg/PLOSOne_formatting_sample_main_body.pdf and

https://journals.plos.org/plosone/s/file?id=ba62/PLOSOne_formatting_sample_title_authors_affiliations.pdf

Response: *The file name is according the Plos One guidelines.*

2.Thank you for stating the following in the Acknowledgments Section of your manuscript:

"The authors would like to thank the Instituto Maua de Tecnologia for funding this work."

We note that you have provided funding information that is not currently declared in your Funding Statement. However, funding information should not appear in the Acknowledgments section or other areas of your manuscript. We will only publish funding information present in the Funding Statement section of the online submission form.

Please remove any funding-related text from the manuscript and let us know how you would like to update your Funding Statement. Currently, your Funding Statement reads as follows:

"The author(s) received no specific funding for this work."

Please include your amended statements within your cover letter; we will change the online submission form on your behalf.

Response: *The sentence was removed and the information included in the conver letter and the Plos One system as required.*

3. We noted in your submission details that a portion of your manuscript may have been presented or published elsewhere. (preprint on Research square.)

Please clarify whether this [conference proceeding or publication] was peer-reviewed and formally published. If this work was previously peer-reviewed and published, in the cover letter please provide the reason that this work does not constitute dual publication and should be included in the current manuscript.

Response: *There is a preprint version of this manuscript as indicated in the cover letter.*

4. We note that Figure 9 in your submission contain map images which may be copyrighted. All PLOS content is published under the Creative Commons Attribution License (CC BY 4.0), which means that the manuscript, images, and Supporting Information files will be freely available online, and any third party is permitted to access, download, copy, distribute, and use these materials in any way, even commercially, with proper attribution. For these reasons, we cannot publish previously copyrighted maps or satellite images created using proprietary data, such as Google software (Google Maps, Street View, and Earth). For more information, see our copyright guidelines: http://journals.plos.org/plosone/s/licenses-and-copyright.

We require you to either (1) present written permission from the copyright holder to publish these figures specifically under the CC BY 4.0 license, or (2) remove the figures from your submission:

Response: *the maps were generated using a python library Plotly. The guidelines to cite this library was obtained at https://plotly.com/chart-studio-help/citations/*

Response to Reviewer #1 comments

The topic is interesting however the study has few flaws and requires minor revisions in order to improve the study in the following ways:

Graphical/Tabular performance analysis of the proposed work with other existing ML models

Response:

To complement the work in this sense, we have included some references that support the choice of a dynamic model over other strategies. An extensive study between empirical models, statistical inferences and dynamic models is referenced in the text to guide the reader. Two other works aiming at the prediction and improvement of the model are cited in the conclusion relating to future works. We understand that these references, added to the work and additional material made available to readers at the address, are sufficient to support the choice and enable modifications to be implemented from the open source code available.

Inclusion of limitation of the model

Response: *A discussion was included at line and in the conclusion we point all the improvements of the model serving as a way to understand its limitation We reinforce that the data do not allow a prediction of the total number of cases and only the cases that will seek medical assistance, which we believe to be the main limitation of the model. An extensive discussion is presented in the documentation open to the public, which includes a study of other epidemics (influenza, for example) and other countries with different characteristics: China, Germany and Italy.*

Conclusion may be further elaborated (page 18)

Response: *new references and arguments were proposed to support the conclusion and future works.*

Check on the repetition of the same line twice where Δt and P is mentioned (2nd paragraph, page 9)

Response: *it was corrected.*

Response to Reviewer #2 comments

The work presents a new proposal for estimating health system use for COVID-19 cases for the whole Brazil. The paper seems good, well-described and structured. However, the comments and the requirements given below should be addressed before accepting the paper

There are some grammar and spelling mistakes in the paper, the language quality should be enhanced.

Response: *the paper was reviewed.*

2 In section 2 on page 3 (Section the model), the sentence (where Δt is the sample time of the data-sets, and P is the total population that should be considered for each case study) is repeated. The repeated sentence must be removed.

Response: *It was corrected*.

3 All symbols in equations should be clarified.

Response: *all symbols were reviewed.*

4 The paper needs to add a review section (Related works) and include some well-established works on predicting COVID-19 that done until now.

Response: *new references were included putting in evidence the model limitation indicating new features and other prediction models and the respective results in the same type (dynamics models).*

5 Add some suggestions for further works and change the title of "conclusion" section to "conclusion and future directions".

Response: *it was included at the conclusion.*
