# Supplementary material for: Predicting COVID-19 in very large countries: The case of Brazil
Source: PLoS One. 2021 Jul 1;16(7):e0253146. doi: 10.1371/journal.pone.0253146 (PMC8248665; doi:10.1371/journal.pone.0253146)
Supplement: Attachment — Submitted filename: Response to Reviewers.pdf [file pone.0253146.s002.pdf]

Santo André, 14 de maio de 2021

MAUÁ

Vanderlei C. **Parro**

Full professor

Tél : +55 (11)4239 3167

vparro@maua.br

Dear Sirs,

Dear Editor and reviewers, Thank you for giving us an opportunity to revise our manuscript entitled “Predicting COVID-19 in very large countries: the case of Brazil”. We found the reviewers’ comments/feedback very helpful in improving the manuscript and we have revised it accordingly. Please find attached the revised manuscript. This work was supported by Mauá Institute of Technology. We declare that all authors have no conflicts of interest. The manuscript has not been published in any other journal. There is a preprint version at: https://www.researchsquare.com/article/rs-79708/v1. Our point-by-point comments on the suggested revisions are below.

Thank you for your consideration of this manuscript.

To:

Thippa Reddy Gadekallu
Academic Editor
PLOS ONE

Sincerely,

V.C.Parro

IMT

Praça Mauá,01 – CEP 09580-900

São Caetano do Sul - São Paulo

www.maua.br

1 Response to Reviewers

1. **The English language in the paper is loose in some instances. The paper needs a thorough proofread:** the review was redone with the indicated orientation.
2. **There are several long sentences in the paper. The authors can break them down into smaller sentences:** the review was redone with the indicated orientation.
3. **List out the main contributions of the paper.:** a paragraph summarizing the contributions was inserted in the introduction. In our understanding, the demonstration that a SIRD model for the whole of Brazil does not represent the actual observed behavior and that, a linear combination of models of the same nature, can represent the behavior of the whole country brings the main contribution. Based on this contribution, the extraction of parameters for public management, as well as predictions that can be reconciled, are equally important contributions.
4. **Some of the recent works such as the following can be discussed in the paper: "Deep learning and medical image processing for coronavirus (COVID-19) pandemic: A survey, An Incentive Based Approach for COVID-19 planning using Blockchain Technology":** Both works were referenced in the context of the introduction, expanding the illustration of the use of machine learning to confront CoViD and how the model can be used in a broader context of monitoring individuals, cases, tests and vaccines.
5. **Discuss about the limitations of the present work:** The limitations are described in the conclusion that we emphasize the explicit non-compliance with vaccination as well as the fact that the algorithm has not been validated considering subsequent waves of CoViD. We emphasize that all documentation is available for use openly, as well as data. In addition, obviously the interest of the authors in the use of the work by other researchers and interested parties.

One extra author was included in this version of the paper. The previous version had acknowledged discussions with F. Pait. Due to his involvement in preparing the revised version, he is now included as a coauthor.
